# Supplementary material for: Subcellular Niche Segregation of Co-Obligate Symbionts in Whiteflies
Source: Microbiol Spectr. 2022 Dec 22;11(1):e04684-22. doi: 10.1128/spectrum.04684-22 (PMC9927142; doi:10.1128/spectrum.04684-22)
Supplement: Reviewer comments [file reviewer-comments.pdf]

AMERICAN SOCIETY FOR MICROBIOLOGY | Microbiology Spectrum

Subcellular niche segregation of co-obligate symbionts in whiteflies

Akiko Fujiwara, Xian-Ying Meng, Yoichi Kamagata, and Tsutomu Tsuchida

Corresponding Author(s): Tsutomu Tsuchida, Toyama Daigaku

**Review Timeline:**	Submission Date:	November 23, 2022
	Accepted:	December 9, 2022

Editor: John Chaston

Reviewer(s): The reviewers have opted to remain anonymous.

Transaction Report:

(Note: With the exception of the correction of typographical or spelling errors that could be a source of ambiguity, letters and reports are not edited. The original formatting of letters and referee reports may not be reflected in this compilation.)

DOI: https://doi.org/10.1128/spectrum.04684-22

December 9, 2022

Dr. Tsutomu Tsuchida
Toyama Daigaku
Faculty of Science, Academic Assembly
3190 Gofuku
Toyama, Toyama 930-8555
Japan

Re: Spectrum04684-22 (**Subcellular niche segregation of co-obligate symbionts in whiteflies**)

Dear Dr. Tsutomu Tsuchida:

Your manuscript has been accepted, and I am forwarding it to the ASM Journals Department for publication. Thank you for considering Microbiology Spectrum as a venue for these findings. You will be notified when your proofs are ready to be viewed.

The ASM Journals program strives for constant improvement in our submission and publication process. Please tell us how we can improve your experience by taking this quick Author Survey.

As an open-access publication, Spectrum receives no financial support from paid subscriptions and depends on authors' prompt payment of publication fees as soon as their articles are accepted. You will be contacted separately about payment when the proofs are issued; please follow the instructions in that e-mail. Arrangements for payment must be made before your article is published. For a complete list of **Publication Fees**, including supplemental material costs, please visit ourwebsite.

ASM policy requires that data be available to the public upon online posting of the article, so please verify all links to sequence records, if present, and make sure that each number retrieves the full record of the data. If a new accession number is not linked or a link is broken, provide production staff with the correct URL for the record. If the accession numbers for new data are not publicly accessible before the expected online posting of the article, publication of your article may be delayed; please contact the ASM production staff immediately with the expected release date.

Corresponding authors may join or renew ASM membership to obtain discounts on publication fees. Need to upgrade your membership level? Please contact Customer Service at Service@asmusa.org.

Thank you for submitting your paper to Spectrum.

Sincerely,

John Chaston
Editor, Microbiology Spectrum

Journals Department
American Society for Microbiology
1752 N St., NW
Washington, DC 20036
E-mail: spectrum@asmusa.org

Supplemental Movie: Accept
Supplemental Material: Accept
